# The Relationship between Executive Function and Obesity in Children and Adolescents: A Systematic Literature Review

**DOI:** 10.1155/2013/820956

**Published:** 2013-02-21

**Authors:** Kaela R. S. Reinert, Eli K. Po'e, Shari L. Barkin

**Affiliations:** ^1^Medical Student at the Medical University of South Carolina, 169 Ashley Avenue, Charleston, SC 29403, USA; ^2^Department of Pediatrics, Vanderbilt University Medical Center, 2146 Belcourt Avenue, 2nd Floor, Nashville, TN 37212, USA; ^3^Diabetes Research and Training Center, Vanderbilt University School of Medicine, 1211 Medical Center Drive, Nashville, TN 37212, USA

## Abstract

The objective of this paper is to examine the relationship between the development of executive function (EF) and obesity in children and adolescents. We reviewed 1,065 unique abstracts: 31 from PubMed, 87 from Google Scholar, 16 from Science Direct, and 931 from PsycINFO. Of those abstracts, 28 met inclusion criteria and were reviewed. From the articles reviewed, an additional 3 articles were added from article references (*N* = 31). Twenty-three studies pertained to EF (2 also studied the prefrontal and orbitofrontal cortices (OFCs); 6 also studied cognitive function), five studied the relationship between obesity and prefrontal and orbitofrontal cortices, and three evaluated cognitive function and obesity. Inhibitory control was most often studied in both childhood (76.9%) and adolescent (72.7%) studies, and obese children performed significantly worse (*P* < 0.05) than healthy weight controls on various tasks measuring this EF domain. Although 27.3% of adolescent studies measured mental flexibility, no childhood studies examined this EF domain. Adolescents with higher BMI had a strong association with neurostructural deficits evident in the OFC. Future research should be longitudinal and use a uniform method of EF measurement to better establish causality between EF and obesity and consequently direct future intervention strategies.

## 1. Introduction

In the past forty-five years, the incidence of obesity in the pediatric population in the United States has more than tripled, with approximately one in three children aged 2–19 classified as overweight (body mass index (BMI) 85–94% for age and sex) or obese (BMI ≥95% for age and sex) [1]. While it is clear that obesity correlates with negative health outcomes such as hypertension and Type II Diabetes, mounting evidence now links obesity to poorer adult cognitive functioning [2–4]. Specifically, studies demonstrate an association between adult obesity and decreased cortical gray matter volume with poorer performance on cognitive assessments [2, 3, 5–8]. One theory is that decreased cognition results from the hypertensive effects, often comorbidity with obesity [9]. Elias and colleagues determined that hypertension and obesity together have a cumulative negative relationship with cognition; however they also predict poorer cognition independently [10]. The interplay between obesity and brain function relates to executive function (EF), which refers to self-regulatory cognitive processes that are associated with monitoring and controlling both thought and goal-directed behaviors [4, 11]. There are several domains of EF, including (1) inhibitory control (suppression of actions that are inappropriate in a given context and that interfere with a goal-driven behavior), (2) attention (the ability to maintain a consistent behavioral response during continuous and repetitive activity) and the closely related concept of mental flexibility (disengagement of an irrelevant task set and subsequent engagement of a relevant task set despite interference and/or priming), (3) reward sensitivity (the relative dominance of the behavioral activation system driving motivated behavior associated with risk-taking behavior), and (4) working memory (active maintenance and flexible updating of goal/task relevant information with limited capacity). Because the vast majority of studies investigating the relationship between obesity and varying domains of executive function are cross-sectional rather than longitudinal, there is a question of directionality in the relationship [4, 12].

Predisposition to obesity could include a dysregulation of specific limbic neural circuits connected with the orbitofrontal cortex [9, 12], given that these limbic circuits and the orbitofrontal cortex are associated with the inhibitory dimension of EF. In prior studies, the orbitofrontal cortex volume is positively associated with high quality food choices and performance on measures of executive function [13]. Rothemund and colleagues compared activity levels of the dorsal striatum, associated with reward anticipation and habit learning, and the OFC in obese versus lean adults with functional magnetic resonance imaging (fMRI) [14]. Obese adults exhibited higher levels of activation in both the dorsal striatum and OFC [14]. Stice demonstrated comparable results in adolescents [15]. This comparative hyperactivation of the OFC in obese individuals could suggest that this cortical area is working harder to suppress chronically hyperactive appetite-stimulating areas [16].

Alternatively, obesity could induce development of neural dysfunction. In a longitudinal study, Sabia and colleagues examined the extent to which lifetime obesity influences midlife cognition [17]. BMI measurements were attained from subjects during early adulthood (mean age = 25), early midlife (mean age = 44), and late midlife (mean age = 61). Results revealed that being obese at 2-3 of these time points was associated with lower scores of executive function, even after adjusting for age, sex, and education difference. Poorer executive function was also associated specifically with a large increase in BMI between early and late midlife [17]. In a separate longitudinal study, Gustafson determined that risk of atrophy of the temporal lobe increased 13–16% per 1.0 kg/m^2^ increase in BMI [18]. However, the study did not find significant evidence of atrophy in the frontal, occipital, or parietal lobes [18].

If obesity does have a detrimental effect on the brain, it would stand to reason that it is important to prevent the development of obesity during a critical period of brain development, particularly during the development of executive function which has been closely correlated with academic success, social function, and emotional control [19–21]. For example, working memory and inhibition are associated with achievements in English, mathematics, and science for 11- and 12-year-old children [19]. Several studies have shown that children aged 3–5 undergo significant and rapid development of executive function which continues to mature into adolescence [22–26]. The prefrontal cortex so closely associated with executive function may be the last region of the brain to mature, and each dimension of EF (e.g., inhibition, shifting, and working memory) may have its own developmental trajectory and timeline [20, 25, 26]. Therefore, executive function is quite vulnerable to a stressor such as obesity during childhood. 

There is an obvious need for novel prevention and intervention strategies to curb the childhood obesity epidemic. Enhancing our understanding of neural mechanisms associated with pediatric obesity could direct these future strategies [27]. As evidenced above, there is a relationship between BMI and executive function in adults, but there still remains a question of causality. In children and adolescents, the relationship between these two variables is less clearly established. Therefore to guide future pediatric obesity prevention efforts, this systematic literature review will seek to answer the question, “What is the relationship between executive function and obesity in children and adolescents?” To better understand the potential mechanisms that might link EF and obesity, we also included an assessment of the neurostructural published studies and obesity. Lastly, we collected data regarding how frequently cognitive function was evaluated, given that it is considered a possible mediator of executive function.

## 2. Methods

### 2.1. Literature Search Strategy

We conducted a systematic literature review, with eligibility criteria and search strategy created a *priori * and based on *The Cochrane Handbook of Systematic Reviews* [28]. The databases searched included PubMed, Google Scholar, PsycINFO, and Science Direct. A broad search was conducted using the keywords: executive function, children, and obesity, to determine the central executive function (EF) domains evaluated in current studies. Inhibitory control, attention/mental flexibility, reward sensitivity, and working memory were the four recurrent domains determined. As a result of this preliminary search, the final keywords utilized for the investigation were executive function, inhibitory control, attention, mental flexibility, reward sensitivity, working memory, cognitive function, prefrontal cortex, orbitofrontal cortex, BMI, obesity, adolescence, pre-school, and healthy children.

The search was conducted by one reviewer (K. R. S. Reinert). The reviewer assessed the abstract for inclusion criteria and then examined each full-text report for quality assessment and data extraction. Inclusion criteria included English language ages 2–18 all races, ethnicities, and genders, healthy participants, excluding obesity and randomized controlled trials (RCTs), meta-analysis, longitudinal studies, cross-sectional studies, prospective and retrospective review studies and those published since the year 2000. Failure to meet at least one of these criteria resulted in study exclusion. When in doubt, the complete paper was screened using the same criteria. Studies excluded measured EF performance in children who had additional diagnoses in addition to being overweight (e.g., ADHD, Type II Diabetes, and sleep apnea), instituted an intervention which altered EF performance (e.g., aerobic exercise), or measured fitness rather than obesity. Studies were organized according to variables evaluated: executive function (EF) orbitofrontal (OFC) and prefrontal (PFC) cortices, or cognitive function. For studies included pertaining to EF, information extracted during the literature review included BMI or BMI percentile of subject groups, age of pediatric population studied, method of measurement of EF, and comparative differences in EF task performance. For studies included pertaining to OFC or PFC imaging, information extracted during the literature review included weight classification of subjects, age of pediatric population studied, method of OFC and PFC imaging, and comparative differences in OFC and PFC structure and function. For studies pertaining to cognitive functioning, information extracted included whether this variable was measured as a mediator of EF or independent of EF, method of cognitive function assessment, and results.

## 3. Results

We reviewed 1,065 unique abstracts: 31 from PubMed, 87 from Google Scholar, 16 from Science Direct, and 931 from PsycINFO. Of those abstracts, 28 met inclusion criteria and were reviewed. From the articles reviewed, additional 3 articles were added from article references (*N* = 31). Twenty-three studies pertained to executive function (2 also studied the prefrontal and orbitofrontal cortices; 6 also studied cognitive function), five studied the relationship between obesity and prefrontal and orbitofrontal cortices, and three evaluated cognitive function and obesity. 

## 4. Executive Function and Obesity

Of the 31 abstracts which met inclusion criteria, 23 examined executive function (EF).  Table 1 summarizes the results, examining each of the EF domains measured, specifying the age of participants, indicating the method of EF domain assessment, and relating the association of each EF domain with obesity.  Table 2 illustrates the relative distribution of the total number of studies conducted in childhood versus adolescent pediatric subjects, specific to EF domains.

### 4.1. Childhood

Thirteen articles examined our research question with children aged 2–12 years old. The 4 EF domains examined were inhibitory control, reward sensitivity, attention, and working memory (Table 1). Inhibitory control was most often examined (76.9%) in the childhood studies (Table 2). A range of tasks measured this EF domain and included Delay of Gratification Task, Self-Control Task, Children's Behavior Questionnaire, Classroom Engagement, Social Behavior Questionnaire, Go-No Go Task, Behavioral Rating Inventory of Executive Functioning (self-report), and Incompatibility Task of Attention Assessment Battery. Despite the great variability in assessment methods, the studies together demonstrate an association between higher childhood BMIs for age and poorer performance on inhibitory control tasks [29–36]. Additionally, some studies demonstrated the predictive value of inhibitory control at a young age: poorer performance at a young age (2–7 years) predicted a higher BMI at a later age (5.5–15 years) (refer to Table 1). Conversely, a better inhibitory performance at age 6 predicted a healthier BMI at age 10-11 [31]. 

The remaining 3 alternative EF domains (attention, reward sensitivity, and working memory) each had few representative studies and therefore corresponded to only 38.4% of the total number of studies examining the relationship between EF and obesity in the pediatric population. Obese females were shown to have poorer ability to focus attention compared to healthy females, but this relationship was not evidenced in males [37]. Reward sensitivity was associated with impulsivity and proven to significantly predict BMI indirectly through propensity to overeat, assessed by parent-reported Child's Eating Behavior Questionnaire (*P* < 0.001) [38]. Huh et al. elucidated five latent classes of obesity risk in 4th grade children based on their behavior: [39] (1) high sedentary behavior, high fat/high sugar (HF/HS) intake, and weight conscious; (2) high sedentary behavior, HF/HS intake, and not weight conscious; (3) dieting without exercise, weight conscious; (4) active, healthy eating; (5) low healthy, snack food, inactive, and not weight conscious. They noted a significant association between classification and child weight status (*P* < 0.001) [30]. Specifically, Riggs et al. determined that youth who were classified as class 1, 2, or 3, demonstrated significantly poorer working memory than children in the two healthier classes (*P* < 0.001) [30]. 

### 4.2. Adolescence

Eleven articles examined our research question with adolescents aged 13–18 years old. There were 4 main EF domains evaluated: inhibitory control, attention/mental flexibility, reward sensitivity, and working memory. Inhibitory control was assessed in most of these (72.7%). A range of tasks measured inhibitory control and included Go-No Go Task, Incompatibility Task of Attention Assessment Battery, Stop Signal Task, Iowa Gambling Task, Stroop Task, Five-Digit Test, and Computerized Cognitive Test Battery. Obese individuals demonstrated less inhibitory control than healthy weight adolescents, and poor performance on inhibitory control tasks was associated with smaller orbitofrontal cortex volume in obese teenagers (refer to Table 1). One study which aimed to evaluate inhibitory control suggested that the variability of responses to their tasks indicated lapses in attentional ability rather than inhibitory control [34].

The second most examined EF domain in adolescents was both mental flexibility and attention, which were evaluated by 27.3% of the adolescence studies (Table 2). Included studies used the following tasks: Trail-Making Test, Wisconsin Card-Sorting Test, Computerized Cognitive Test Battery, Five-Digit Test Switching, Color-Word Interference Test Stroop, and D2 Attention Endurance Test (refer to Table 1). For every one of these tasks, obese adolescents performed worse than healthy weight participants (Table 1). Within obese participants, BMI was inversely related to performance on the Color-Word Interference Test Stroop (Table 1). Working memory and reward sensitivity had only one representative study each (9.0%) (Table 2). Similar to attention/mental flexibility, working memory performance of obese adolescents was significantly worse than that of the healthy weight controls, even after controlling for IQ (110.7 ± 13.3 versus 99.4 ± 13.8;  *P* < 0.001) [12]. 

### 4.3. Cognitive Function

Nine of the 31 articles (29.0%) included in this paper included cognitive function as well as EF. A third of these studies examined cognitive function as a mediator of executive function and therefore controlled for the subject's intelligence quotient or academic performance when determining the association between EF and obesity. All of these studies noted an inverse relationship between excess weight and performance on EF tasks even after controlling for general cognitive function. The remaining two-thirds of articles examined the association between general cognitive function and obesity. Half of these studies found a null relationship. One study examined this in children using the planning subtest of the Cognitive Assessment System, which pertains to aspects of EF including strategy generation and application, self-regulation, intentionality, and utilization of knowledge. Performance was significantly inversely related to both waist girth (*P* < 0.05) and body fat (*P* < 0.01) of children 7–11 year olds [53]. 

## 5. Brain Imaging and Obesity

Brain imaging indicates specific areas of the brain where different domains of EF function, specifically the dorsolateral prefrontal cortex (DLPFC) and the orbitofrontal cortex (OFC) [4, 54, 55]. In our systematic literature review, we found 7 studies which examined the relationship between these areas of interest in the brain and obesity in adolescents (refer to Table 3). Two of these studies had also examined EF. All 7 of the included studies evaluated the OFC, while only 3 assessed the DLPFC. The literature research yielded no data available for the pediatric population under the age of 9 years old. Three studies examined the reactivity of the DLPFC and OFC in response to food images using functional magnetic resonance imaging (fMRI) (Table 3). Obese subjects demonstrated significantly (*P* < 0.001) greater activation of the PFC (before meal) and significantly (*P* < 0.001) less reduction of activation of the PFC (aftermeal) compared to healthy weight controls [36]. Davids et al. also found that obese subjects had hyperactivity in the DLPFC compared to healthy weight controls in response to food cues (left DLPFC, *T* = 3.23, *P* < 0.001; right DLPFC, *T* = 2.65, *P* < 0.006), but did not explicitly control for pre- and after meal states [56]. The OFC of obese subjects was also highly activated in obese subjects after meal compared to healthy weight controls (*P* < 0.001) [36]. Batterink et al. more directly evaluated the relationship between EF and obesity by using fMRI to evaluate the OFC, while female participants performed a Go-No Go Task, a classic test of inhibition [46]. This behavioral task presents stimuli in a continuous stream, and subjects perform a binary decision with each stimulus. One stimulus requires a motor response (go), while the other requires the withholding of a response (no go). Body mass index was negatively correlated with OFC activation during performance of the task (*P* = 0.05) [46]. Maayan et al. also combined imaging analyses with performance of a Go-No Go Task and used magnetic resonance imaging to significantly negatively correlate subject performance with individual OFC volume (32.3 ± 3.68 versus 33.3 ± 3.99; *P* = 0.005), thereby linking higher BMI with poorer performance on inhibitory control tasks and smaller OFC grey matter volumes [12]. This indicates that neurostructural deficits associated with obesity and exhibited in behavioral cognitive tasks are apparent by the age of adolescence.

Burger and Stice correlated the dietary restraint score of subjects to their activation of the OFC and DLPFC in response to milkshake anticipation, milkshake receipt, and food images. Elevated activity of right OFC (*r* = 0.57) and bilateral DLPFC (right, *r* = 0.49; left, *r* = 0.55) in subjects in response to milkshake receipt was positively correlated with restraint scores. This relationship suggests hypersensitivity to reward during ingestion of food, which could potentiate risk for obesity. There was no correlation of frontal cortical area activity in neither response to milkshake anticipation nor in response to food images.

## 6. Discussion

As we strive for progress in the development of interventions to curb the pediatric obesity epidemic, we review what we know in order to direct us towards what we need to know. Many studies have revealed the interplay between executive function (EF) and obesity in the adult population; however, few studies demonstrate the extent to which this relationship exists in children and adolescents. We examined 31 studies which met inclusion criteria, and 23 of these evaluated at least one of the 4 common EF domains in our population of interest. In both childhood and adolescence, inhibitory control is associated with BMI and with less control associated with higher BMIs. Four of the studies implicated a predictive nature related to inhibitory control. Poor performance on inhibitory control tasks at age 2 was correlated with higher BMI at age 5.5 even after controlling for age 2 BMI [40]. Poor performance at ages 3 and 5 correlated with higher BMI up to at least the age of 12 and the most rapid weight gain 3–12 years; however, this study did not control for baseline BMI [32]. Poor performance at age 7 significantly predicted BMI at each subsequent time point of ages 7 (*P* < 0.001), 9 (*P* < 0.001), 11 (*P* < 0.001), 13 (*P* < 0.001), and 15 (*P* < 0.001), but this study only examined Hispanic female children [35]. Better performance at age 6 significantly correlated with a healthier weight at ages 9-10, even after controlling for baseline BMI, sports participation, child cognitive skills, and preexisting family characteristics (*P* = 0.030) [31].

The orbitofrontal cortex correlates with inhibitory behavior as measured by functional magnetic resonance imaging (fMRI) and magnetic resonance imaging (MRI). The studies identified through this systematic literature review revealed that both the function and the anatomy of the OFC were altered as evidenced by fMRI and MRI data (see Table 3). However, it is important to note that this relationship was only investigated in youth aged 9 and older. Thus, it isnot clear when the structure and the function were initially altered or if this could be reversed. While obese adolescents showed a decreased level of activation of OFC before meal versus healthy weight controls, they showed elevated OFC activity after meal, functioning inversely to the patterns seen in the lean controls. This could suggest the development of resistance to signaling in the obese adolescent. Before meal the obese adolescent does not demonstrate an inhibitory control signal as strongly as a healthy weight subject, but after meal the obese adolescent shows an elevated inhibitory signal compared to the healthy weight subject, showing a compensatory effect in which the brain ramps up its signaling in attempt to produce a “stop” behavior. Additionally, Batterink et al. correlated higher BMI with a greater number of errors in an inhibitory control behavioral task [46]. Maayan et al. contributes to this finding by revealing that there is a negative correlation between BMI, overall performance on an inhibitory control behavioral task, and OFC gray matter volume [12]. This indicates that there is a difference in neuroanatomy of obese individuals that is detectable behaviorally by the early age of 15 years [12]. This difference in orbitofrontal volumes is especially important during the adolescent years, when the patterning of activity in the OFC of adolescents resembles that of children, and the nucleus accumbens (reward center) patterning of activity resembles that of adults, which serves to predispose adolescents to risk-taking behavior with little self-regulation [59].

In addition to inhibitory control alterations, this paper found differences in the EF domains of attention/mental flexibility, reward sensitivity, and working memory, associated with obesity in both child and adolescent populations. Unfortunately, each of these domains and age groups had few representative studies. In addition, the studies that evaluated multiple domains disagreed about the validity of the differences evidenced in each. For example,Verdejo- García et al. studied adolescents aged 13–16 and found significant differences in obese versus healthy weight subjects within the EF domains of inhibition and mental flexibility [45]. However, the study did not find significant differences in the EF domains of working memory, planning, and reasoning. In contrast, Cserjési et al. found significant differences in working memory as well as attention, but not for intelligence or verbal fluency [51]. The reason for contrary findings among studies may be due to variability of testing method, as evidenced in Table 1. Despite the advantage that the studies included in this paper examined different age ranges, this was confounded by the variability among EF assessment method, even when evaluating the same EF domain. Future research should aim to use a more uniform assessment method, to allow for better comparison of findings and the formation of a more accurate account of the relationship that exists between obesity and executive function in youth. The National Institutes of Health has recently developed an Executive Functioning toolkit to create clearer consistency of methodology and assessment.

This systematic review reveals a consistent inverse association between obesity and executive function in children and adolescents. Therefore, it is imperative to first determine the directionality of this association. This could be accomplished by longitudinal assessments that apply uniform measurement approaches to assess the executive function of participants. Baseline measurements of children's BMI and performance on EF tasks can serve to confirm the existence of a negative correlation between these two variables, while later time points can examine how the EF changes overtime in accordance with changing BMI. Given that cognitive function may be a mediator of executive function, future studies should consider controlling for the intelligence quotient or baseline academic success of participants when evaluating EF. If it is determined that executive dysfunction could predispose a child to obesity, this would inform early intervention strategies. Additional studies would need to establish the extent of decline exhibited as children and adolescents develop and the possibility of reversing the dysfunction. It is important to note that physical activity has been associated with improving EF in children and therefore should remain an integral part of any obesity intervention strategy [53]. This is especially crucial considering that EF is so intimately associated with academic performance [60]. Conversely, if it is determined that lower EF abilities predispose a child to obesity, prevention and intervention strategies should focus on the early improvement of the child's executive function in addition to the teaching of healthy behaviors. 

This, too, is possible. Riggs et al. emphasized changing child impulse control, decision making, and social competence in an intervention strategy [30]. The study found that children demonstrated significant changes in positive attitude towards both self-regulation and appetitive behavior. Children even exhibited positive changes in actual food choices and television viewing patterns [30]. Pauli-Pott et al. studied children aged 7.5–15 years and found that performance on inhibitory control tasks was positively associated with the ability to lose weight during a 1-year intervention program, but this association pertained more to adolescents than younger children [47]. Taken together, these studies evidence the idea that perhaps a successful intervention program would focus on the improvement of executive function, with particular emphasis on the inhibitory control domain, *in addition* to healthy behaviors such as eating well and exercise. Perhaps it is not sufficient to focus solely on how children eat and move, but it is necessary to build on how they think.

## 7. Limitations

This paper included only the published literature; therefore, unpublished work was excluded. This type of publication bias could lead to an incomplete understanding of the current state of knowledge about the association between EF and childhood/adolescent obesity.

## 8. Conclusion

Future research examining the link and directionality between EF and childhood obesity should be longitudinal rather than cross-sectional and use a uniform method of EF measurement to direct future intervention strategies. 

## Figures and Tables

**Table 1:** Association between EF and obesity in childhood versus adolescence.

EF Domain	Participant age	Measure used	Findings	Source
Childhood				

	2–5.5 yrs	Delay of gratification task	2 yrs performance predictive of 5.5 yrs obesity when considered with emotional regulation	Graziano et al. (2010) [29, 40]
	3–12 yrs	Self-control (age 3) Delay of gratification (age 5) Children's Behavior Questionnaire (age 5)	Children with poorer performance at ages 3 and 5 had significantly higher BMI at all subsequent time points and had the most rapid gain in BMI 3–12 yrs	Francis and Susman (2009) [32]
	6 yrs	Classroom engagement Social behavior questionnaire	Better performance at age 6 correlated with healthier weight in 4th grade	Piché et al. (2012) [31]
(I) Inhibitory control	5–15 yrs	Child behavior questionnaire	Subjects with low inhibitory control at age 7 tended to have higher BMIs at all follow-up measurements and experienced greater weight gain at age 7–15	Anzman and Birch (2009) [35]
	7–9 yrs	Go-No Go Task	Higher BMI correlated with poorer performance	Kamijo et al. (2012) [33, 41]
	8-9 yrs	Behavioral Rating Inventory of Executive Function (self-reporting)	Highly sedentary children who were not weight conscious and consumed high fat and high sugar snacks exhibited less inhibitory control than children who were active and consumed fruits and vegetables. EF proficiency negatively correlated with substance use, high-calorie snack food intake, and sedentary behavior, while positively associate with fruit and vegetable intake as well as out-of-school physical activity	Riggs et al. (2012) [30, 42]
	8–11 yrs	Go-No Go and Incompatibility Tasks of Attention Assessment Battery	High impulsivity linked to higher body weight	Pauli-Pott et al. (2010) [34]
	8–12 yrs	Delay of Gratification Task (nonfood reward)	O/OW less likely to delay gratification than HW and overweight* peers	Bruce et al. (2011) [27]
		Go-No Go Task	O/OW had lower response accuracy for No Go component of task than healthy weight controls	Kamijo et al. (2012) [41]

(II) Attention	1–6 yrs	Attention span persistence	Among boys, greater persistence at age 1 associated with reduced standardized weight gain and reduced obesity risk through age 6	Faith and Hittner (2010) [43]
	4–8 yrs	Modified “Bavarian Model” for school entry examinations	O/OW females had greater prevalence of inability to focus attention than HW females (but not males)	Mond et al. (2007) [37]

(III) Reward sensitivity	6–13 yrs	Sensitivity to punishment and sensitivity to reward questionnaire for children	Performance significantly predicts BMI indirectly through overeating	Van den Berg et al. (2011) [38]

(IV) Working memory	8-9 yrs	Behavioral Rating Inventory of Executive Function (self-reporting)	Children who were highly sedentary and consumed high fat and high sugar foods exhibited poorer working memory and poorer organizational skills than children considered active and who ate fruits and vegetables. EF proficiency negatively correlated with substance use, high-calorie snack food intake, and sedentary behavior, while positively associate with fruit and vegetable intake as well as out-of-school physical activity	Riggs et al. (2012) [30, 42]

Adolescence				

	12–15 yrs	Go-No Go and Incompatibility Tasks of Attention Assessment Battery	Variability of responses and tendency for relationship of body weight and performance to be inverse indicate attentional lapses rather than distinctly inhibitory lapses	Pauli-Pott et al. (2010) [34]
	12–15 yrs	Stop Signal Task	O/OW have less inhibitory control than HW	Nederkoorn et al. (2006) [44]
	13–16 yrs	Iowa Gambling Task	O/OW performed significantly worse than HW controls	Verdejo-García et al. (2010) [45]
(I) Inhibitory control	12–21 yrs	Go-No Go Test Stroop Task Five-Digit Test Computerized Cognitive Test Battery	O/OW showed significantly more false positive responses and shorter reaction time than HW; significant association between disinhibition, OFC volume, and BMI	Batterink et al. (2010) [46]; Maayan et al. (2011) [12]; Verdejo-García et al. (2010) [45]
	7.5–15 yrs	Go-No Go Task Interference task	High impulsivity predicted successful weight loss in adolescents	Pauli-Pott et al. (2010) [47]
	10–14 yrs	The stop task Circle drawing task Opposite worlds task Maudsley Index of Childhood Delay Aversionand Door-Opening Task	Association was found with overweight children and less efficient inhibitory control	Verbeken et al. (2009) [48]
	12–17 yrs	Letter-Number Sequencing Stroop and Iowa Gambling Task	Greater improvement in cognitive inhibitory control skills was associated with greater reductions in BMI	Delgado-Rico et al. (2012) [49]

(II) Attention/Mental flexibility	12–19 yrs	Trail making test Wisconsin card sorting test Computerized Cognitive Test Battery Five-Digit Test-Switching Color-Word Interference Test Stroop D2 Attention Endurance Test	O/OW performed significantly worse than HW on all tasks; BMI inversely related to Stroop-switching performance for O/OW subjects	Lokken et al. (2009) [50]; Cserjesi et al. (2007) [51]; Verdejo-García et al. (2010) [45]; Delgado-Rico et al. (2012) [52]

(III) Reward sensitivity	12–15 yrs	Door-Opening Task	O/OW were more sensitive to reward and kept gambling longer than HW	Nederkoorn et al. (2006) [44]

(IV) Working memory	13–21 yrs	Working memory index of WRAML and Letter-Number sequencing	O/OW performed worse than HW controls	Maayan et al. (2011) [12]

Obese/Overweight (O/OW) versus Healthy Weight (HW): subjects classified as overweight or obese met the criteria of BMI ≥30 kg/m^2^ or >95 percentile for BMI for age and gender; subjects classified as healthy weight met the criteria of BMI <25 kg/m^2^ or within 5–85 percentile for BMI for age and gender.

*Overweight: BMI between 85 and 95%.

**Table 2:** Comparative distribution of each EF domain included in childhood versus adolescence studies.

Function measured	% of total included studies which examine the EF domain in children (% of childhood studies which examine the EF domain)	% of total included studies which examine the EF domain in adolescents (% of adolescence studies that examine the EF domain)	% of total included studies that examine the EF domain
Inhibitory control	43.5% (76.9%)	34.7% (72.7%)	73.9%
Attention	8.7% (15.3%)	13.0% (27.3%)	21.7%
Mental flexibility	0% (0%)	13.0% (27.3%)	13.0%
Reward sensitivity	4.3% (7.7%)	4.3% (9.0%)	8.7%
Working memory	8.7% (3.8%)	4.3% (9.0%)	13.0%
Total studies included	56.5%	47.8%	

**Table 3:** Association between BMI and brain imaging of dorsolateral prefrontal cortex (DLPFC) and orbitofrontal cortex (PFC).

Brain region	Participant age	Measure used	Findings	Source
DLPFC	9–18 yrs	Activation response before meal and after meal to pleasant, neutral, or food images (fMRI)	O/OW showed greater activation in DLPFC (before meal) and less reduction of activation in PFC (after meal) compared to HW in response to food images	Bruce et al. (2010) [36]; Davids et al. (2010) [56]
DLPFC	14–16 yrs	Activation in response to milkshake receipt, milkshake anticipation, and food images (fMRI)	Dietary restraint scores positively correlated with activation in bilateral DLPFC in response to milkshake receipt	Burger and Stice 2011
OFC	9–18 yrs	Activation response before meal and after meal to pleasant, neutral, or food images (fMRI)	O/OW showed greater OFC activation versus HW (after meal)	Bruce et al. (2010) [36]; Davids et al. (2010) [56]
OFC	10–14 yrs	Activation while viewing food and nonfood logos	O/OW had significantly less activation versus healthy weight in response to food logos	Bruce et al. (2012) [57]
OFC	14–16 yrs	Activation response during Go/No-Go Task (fMRI)	Negative correlation between BMI and level of OFC activation during task	Batterink et al. (2010) [46]
OFC	14–16 yrs	Activation in response to milkshake receipt, milkshake anticipation, and food images (fMRI)	Dietary restraint scores positively correlated with activation in right OFC in response to milkshake receipt	Burger and Stice 2011
OFC	14–17 yrs	Activation in response to most and least appetizing food images subject previously rated (fMRI)	BMI correlated positively with activation during initial orientation to food cues and predicted future increases in BMI	Yokum et al. (2011) [58]
OFC	15–19 yrs	Magnetic resonance imaging	BMI negatively correlated with OFC volume and positively correlated with greater number of commission errors	Maayan et al. (2011) [12]
